# Printing Personalized Medicine: 3D Models Bring Better Surgical Outcomes

**DOI:** 10.2196/100950

**Published:** 2026-05-25

**Authors:** Jenna Congdon

**Keywords:** three-dimensional printing, models, anatomic, surgical procedures, operative, patient-specific modeling, airway management, prostheses and implants, precision medicine

## Abstract

Advances in medical 3D printing have opened up new possibilities for surgical training, planning, and decision-making. In this *News and Perspectives* article, JMIR Correspondent Jenna Congdon reports on the use of 3D printing in a pediatric surgical case and examines the broader implications of 3D printing for surgical practice.

**Key Takeaways:**

- 3D-printed anatomical models can reduce risk and support better outcomes for high-risk clinical decisions and surgical procedures.
- Integrating 3D printing technology with medicine enables more precise surgical planning, improved training, and patient-specific care across specialties.
- Despite clear benefits, adoption is limited by cost, insurance coverage gaps, and regulatory barriers.

A multidisciplinary group of physicians—neonatologists, anesthesiologists, and ear, nose, and throat surgeons—pass a 3D-printed model of a baby’s head between them. This meeting has been called prior to the high-stakes delivery of an infant with a complex facial mass that the team fears may block the child’s airway. As they examine the model, the answer becomes clear.

## Technical Skill Meets Human Compassion

Kyle VanKoevering, MD, took an unconventional route to becoming a surgeon. His first degree was in engineering, but that wasn’t enough: “I wanted to figure out that overlap between helping people and being technical.”

A chance job-shadowing opportunity brought him to the operating room. While observing the surgeon at work, VanKoevering recognized his roots: the specialized aspects of surgery drew on the technical skills combined with detailed forethought and structural planning he had learned as an engineer. “I realized this was an opportunity to do both…that blend of engineering in surgery drew me into medical school…It was a very direct way I would be able to help people.”

In the 2010s, when VanKoevering began his surgical career, 3D printing wasn’t new technology, but it wasn’t yet as widely used for medical purposes. His curiosity as an engineer was piqued: how could this technology be used to improve medical and surgical outcomes, particularly in the complex cases he saw as an ear, nose, and throat surgeon?

### Preparing for a Delivery Into the Unknown

In 2015, he got his first opportunity to answer that question: on a routine ultrasound, a large mass was spotted on a fetus’s face, sparking immediate concern over the child’s ability to breathe after birth.

The flat images from computed tomography scans and magnetic resonance imaging couldn’t decisively show whether or not the mass was blocking the baby’s airway. A specialized delivery procedure called an ex-utero intrapartum treatment or EXIT procedure was planned.

During an EXIT procedure, the mother is placed under general anesthesia and a cesarean section is performed, but only the head and neck of the infant are initially delivered. The umbilical cord stays attached to the placenta, providing the newborn with blood flow and oxygen from the mother’s body. This gives the medical team approximately 20‐30 minutes to secure the infant’s airway, typically by inserting an endotracheal tube or tracheostomy. Once the airway is established, the rest of the baby’s body is delivered and the umbilical cord is cut. This delivery method carries higher risks to both parent and baby than a vaginal delivery or standard cesarean section and requires detailed interdisciplinary planning prior to birth.

### 3D Models for a 3D World

This case sparked an idea for VanKoevering: “We live in a 3D world. This is ultimately 3D anatomy, but we’re all looking at this in two dimensional pictures and trying to mentally recreate the 3D picture.” Why not print a 3D model so that the medical team could fully examine the mass?

He led a team to create a 3D model of the infant’s head using images from computed tomography, magnetic resonance imaging, and ultrasound. When they met to discuss the delivery plan, the team could finally see—with collective relief—that the mass did not, in fact, block the infant’s airway.

A standard cesarean delivery was planned instead of the EXIT procedure, with multidisciplinary teams standing by. The child was able to breathe easily, and rather than being under general anesthesia, the mother delivered with only an epidural and was able to hold her child immediately after birth. The 3D-printed model had changed everything.

## Engineering Better Surgical Outcomes

Motivated by this successful outcome, VanKoevering began working to expand the use of 3D printing for complex surgical cases. In 2020, he established the Medical Modeling, Materials and Manufacturing Lab—better known as the M4 Lab—at Ohio State University.

There, biomedical engineers work alongside clinical professionals to develop 3D-printed models for an array of medical uses. Models allow surgeons to study unique patient anatomy before surgery, develop patient-specific prostheses, and train residents on highly technical surgical procedures and challenging intubation scenarios.

### 3D Models as a Surgical Roadmap

The M4 lab isn’t alone in using this technology; hospitals throughout the world use 3D printing to secure better outcomes for their patients. This technology supports safer and more effective surgeries in many disciplines, frequently in orthopedics, pediatric cardiology, neurosurgery, and ear, nose, and throat, among many more.

Operating on the larynx, neck, face, and skull base as specialists like VanKoevering do is often risky; skull base tumors in particular are highly challenging. The bottom of the skull is full of irregular grooves that house delicate brain tissue and the cranial nerves. These nerves control our most basic functions, from facial movement to hearing, taste, vision, smell, swallowing, breathing, heart rate, and speech. 3D-printed models of individual patient anatomy can be brought into the operating room as a tangible, visual guide, allowing surgeons to operate with higher levels of precision and foresight in these delicate cases.

Furthermore, 3D printing can be used to manufacture life-like models, prostheses, and surgical implants that mimic the color and texture of real tissue. These models are used to train new physicians, allowing them hands-on practice without human risk. Patient-specific implants and prostheses allow for faster recovery and fewer negative surgical outcomes.

By helping to preserve both their appearance and their ability to engage in essential activities, these 3D-printed items can allow patients to return to their daily lives feeling more fully themselves. The impact is so powerful that many patients request to keep the models used in planning their surgeries; VanKoevering’s team happily obliges.

**Figure:**
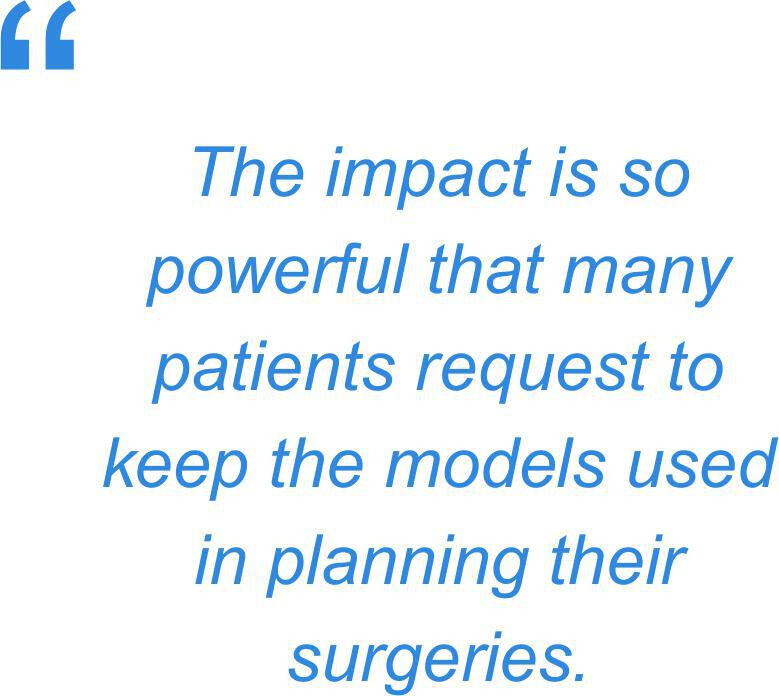
­

## Beyond the OR: Implementation and Barriers

Of course, the broader implementation of 3D printing for medical purposes is not without its barriers. Funding is a perennial hurdle: “We need insurers to buy into seeing the value of this technology, and right now it’s just not there,” VanKoevering says.

Implantable 3D-printed models require rigorous testing and Food and Drug Administration clearance to manufacture, a capability that the M4 lab does not yet have. Other medical facilities struggle with the high costs of starting a 3D printing lab, as well as the integration of the additional training and multidisciplinary coordination that must take place.

Still, personalized surgical care offers abundant benefits to patients and surgeons alike and may lower costs for long-term care if patients experience better overall surgical outcomes.

## Printing Personalized Medicine

VanKoevering is hopeful for the future of 3D printing in medicine. “As I look at this technology, we’ve just continued to look for creative and impactful ways to apply it into personalizing healthcare for patients… that’s really what 3D printing and 3D modeling allows us to do.”

By shifting surgery from a one-size-fits-all approach to a deeply individualized procedure, a simple 3D-printed model becomes much more than a tool meant to enhance surgical precision; it preserves the fundamental human identity of each unique patient.

